# A cautionary note on the power of the test for the indirect effect in mediation analysis

**DOI:** 10.3389/fpsyg.2014.01549

**Published:** 2015-01-12

**Authors:** Tom Loeys, Beatrijs Moerkerke, Stijn Vansteelandt

**Affiliations:** ^1^Department of Data Analysis, Ghent UniversityGhent, Belgium; ^2^Department of Applied Mathematics, Computer Science and Statistics, Ghent UniversityGhent, Belgium

**Keywords:** mediation analysis, power, indirect effect, type I error, confounding, sensitivity analysis

## Abstract

Recent simulation studies have pointed to the higher power of the test for the mediated effect vs. the test for the total effect, even in the presence of a direct effect. This has motivated applied researchers to investigate mediation in settings where there is no evidence of a total effect. In this paper we provide analytical insight into the circumstances under which higher power of the test for the mediated effect vs. the test for the total effect can be expected in the absence of a direct effect. We argue that the acclaimed power gain is somewhat deceptive and comes with a big price. On the basis of the results, we recommend that when the primary interest lies in mediation only, a significant test for the total effect should not be used as a prerequisite for the test for the indirect effect. However, because the test for the indirect effect is vulnerable to bias when common causes of mediator and outcome are not measured or not accounted for, it should be evaluated in a sensitivity analysis.

## Introduction

**Figure 1:**
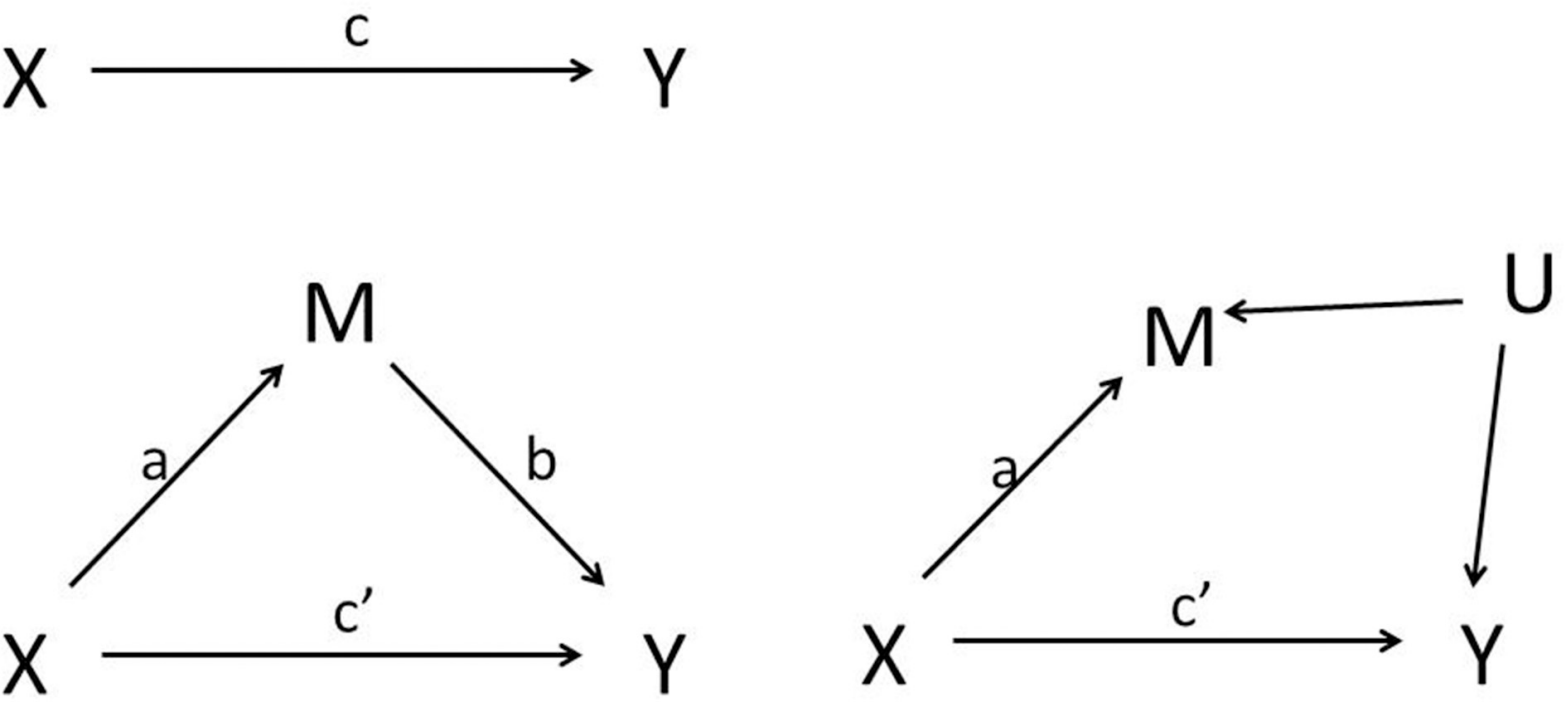
**Left panel: Simple mediation model in which X is the independent variable, M is the mediator and Y is the outcome variable. Right panel: Unmeasured confounding U of the mediator-outcome relationship**.

The Baron and Kenny (1986) description of how mediation can be statistically assessed has led to a massive number of applied publications in the psychological literature over the last 25 years. The left panel of Figure 1 shows the basic components of the classical mediation analysis. Let variable *X* represent the independent variable, a presumed cause of the dependent measure *Y*. The top left panel of the Figure 1 then represents the total effect of *X* → *Y*. The bottom panel represents the mediation model that we will consider throughout this paper. MacKinnon (2008, pp. 38–40) distinguishes two critical parts in this mediation model: the first part is referred to as “action theory,” which describes how the independent variable or intervention *X* changes the mediating variable *M* (the effect captured by the parameter *a*); and the second part as “conceptual theory,” which specifies how the mediator affects the dependent variable (the effect captured by the parameter *b*). Mediation analysis ultimately consists of a simultaneous test of action and conceptual theory. Mediation is thus quantified by the effect of *X* on *Y* through *M*, which is called the indirect effect, while the effect of *X* on *Y* that is not mediated by *M* is referred to as the direct effect.

Assuming linear relationships and continuous variables *M* and *Y*, the direct and indirect effects are typically parameterized using the following set of linear regression models:

(1) $$Y=i_{1}+cX+\varepsilon_{1}$$

(2) $$M=i_{2}+aX+\varepsilon_{2}$$

(3) $$Y=i_{3}+c^{\prime }X+bM+\varepsilon_{3}$$

with *i*_1_, *i*_2_, and *i*_3_ intercepts and ε_1_, ε_2_, and ε_3_ independent mean zero residuals. The parameters *a*, *b*, *c*, and *c*' are estimated using simple linear regression or structural equation modeling (MacKinnon, 2008); they will be denoted â, $\hat{b}$, ĉ, and ĉ', respectively. Under the presumed causal relationships in the left panel of Figure 1 and assuming the models (1), (2), and (3) hold, the estimated parameters ĉ and ĉ' quantify the total and direct effect, while the indirect effect is quantified as â$\hat{b}$. In the above linear setting, the total effect is equal to the sum of the direct and indirect effect, i.e., ĉ = ĉ'+ â$\hat{b}$.

In this paper we will focus on mediation analyses in experiments that randomize the independent variable *X*. The total effect of *X* on *Y* can then be identified without untestable assumptions on the absence of unmeasured common causes (Holland, 1986, 1988). Even so, the estimation of direct and indirect effects may be biased in such randomized experiments. This may happen when a variable other than the independent variable affects both *M* and *Y* and is not controlled for (e.g., because it is unmeasured). This was already made clear in a much less cited predecessor of the Baron and Kenny paper (Judd and Kenny, 1981), repeatedly emphasized over the last decade in methodological papers on mediation analysis (Bullock et al., 2010), and is also the aim of comprehensive structural equation modeling (MacKinnon and Pirlott, 2014). In spite of that, very few applications control for variables that may affect both *M* and *Y*, nor do they discuss how plausible it is to assume the absence of such variables, an assumption often referred to as *no unmeasured confounding of the M-Y relationship*. This is daunting: even when in reality there is no effect of *M* on *Y* at all, and thus no indirect effect, an analysis that ignores common causes of *M* and *Y* may reveal a spurious effect of the mediator on the outcome. One therefore cannot determine based on the observed data whether the indirect effect is (partially) explained by unobserved common causes (Fiedler et al., 2011). One can pro-actively think about potential common causes of mediator and outcome at the design stage, measure those variables and account for them in the analysis; but in practice, it is likely impossible to measure them all.

The greater robustness of tests for the total effect than tests for the mediated effect to the presence of common causes has led researchers to demand, as in the traditional Baron and Kenny approach (MacKinnon, 2008), a significant total effect [i.e., *c* in model (1) being significantly different from zero] as a prerequisite (“step 1”) for conducting a mediation analysis. For instance, one of the earliest critics on this prerequisite mentions “The reviewers of this article had mixed opinions about whether any form of step 1 should be retained. Two believed it should be dropped completely. Another argued for retaining the step because it provides protection against alternative causal models, whereby the associations of (X and M and of) M and Y are spurious” (Shrout and Bolger, 2002). Since then, many scholars have given further pros and cons on the necessity of step 1, but this has not prompted a more unified view and, instead has caused a lot of confusion in the applied mediation literature. Over the last couple of years, however, a clear trend has emerged (Hayes, 2009; Zhao et al., 2010; Rucker et al., 2011; Kenny and Judd, 2014; O'Rourke and MacKinnon, 2014) in favor of dropping the requirement of a significant total effect to assess mediation. This change was largely triggered by simulation studies by Rucker et al. (2011) and more recently by Kenny and Judd (2014) and O'Rourke and MacKinnon (2014), which demonstrated that significant indirect effects can often be detected, even when the total effect is not statistically significant. Researchers who wished to publish their mediation analyses in the absence of a total effect picked up those arguments rapidly (often neglecting the potential threats that were mentioned by those authors), while reviewers and editors may have become more than ever hesitant about the scientific trustworthiness of such analyses (Osborne, 2010; Smith, 2012).

With this paper, we wish to temper some of the enthusiasm around the acclaimed power gain. First, we note that empirical studies have so far focused on the power to detect an indirect effect in the absence of a significant total effect (Rucker et al., 2011; Kenny and Judd, 2014). We assess the type I error of such strategies that test the indirect effect conditional on a non-significant total effect and find it to be inflated. This points toward an increased risk of false positive mediated effects and partially explains the power gain observed by Rucker et al. (2011) and Kenny and Judd (2014). It moreover has immediate implications for applied researchers who adopt the strategy to first test for a total effect, but—if absent—to continue to look for indirect effects, hereby neglecting the potential impact on the risk of false positive findings.

In view of the previous concern, we develop insight into the relative power of the test for the indirect effect vs. the test for the total effect, in the absence of a direct effect, by means of large sample approximations and Monte Carlo experiments. We moreover note that this power gain comes at a cost. First, the estimation of the indirect effect requires stronger modeling assumptions than the estimation of total effects (for e.g., Equation 2 and 3 vs. Equation 1 alone). Misspecification of these assumptions may invalidate the test for indirect effect, and rescind a potential power gain. Second, the test for indirect effect, unlike the test for total effect, requires assumptions about the absence of unmeasured common causes of mediator and outcome. We therefore recommend sensitivity analyses that assess the robustness of mediated effects against violations of the no unmeasured *M*-*Y* confounding assumption. Such confounding may diminish the power of the test for the indirect effect. We explore how strong the violation of this assumption must be, in order for the power to detect the total effect to equal the power to detect the indirect effect. If small violations (which are not unlikely to occur in most studies) quickly lead to such equality in power, then the theoretical power gain of the indirect effect vs. the total effect is of limited practical use. We show that the circumstances under which the power of the test for the mediated effect vs. the power of the test for the total effect is largest, are relatively vulnerable to violations of the no unmeasured *M*-*Y* confounding assumption. We end with a discussion of the implications of our findings and some practical guidelines.

## The relative power of the test for the indirect effect vs. the test for the total effect

Using simulations, Rucker et al. (2011) explored the probability of observing significant effects when the total effect is not significant in a variety of conditions common in psychological research. More precisely, assuming the causal model presented in the left panel of Figure 1, they set the population values of *a* and *b* to 0.4 in all conditions; and varied the population values of *c* (0.2, 0.3, and 0.4) and the sample size *n* (25, 50, 100, 200). The variables *X*, *M*, and *Y* were all normally distributed with variance equal to 1. That is, rather than setting the error variances of *M* and *Y* equal to one in Equation (2) and (3), those were set equal to 1 – *a*^2^ and 1 – *c*'^2^ – *b*^2^ – 2*abc*', respectively. These authors thus use standardized regression coefficients as effect size measures of individuals paths in the mediated effect (MacKinnon, 2008, pp. 80–81). For each combination of conditions, 5000 samples were generated. The authors concluded that “detecting indirect effects in the absence of a total effect can be quite frequent—nearly half of the time—in sample sizes typical of psychological research.” Similarly, Kenny and Judd (2014) presented tables with sample sizes required to achieve 80% power for the test of the null hypothesis that *c* and *ab* each equal zero when the direct effect is zero, and found that, in the presence of such complete mediation, “one might not uncover a statistically significant total effect but might still have sufficient power to detect a significant indirect effect.” In this section, we first argue that the above statements are somewhat deceptive because tests for mediated effects that are being conducted conditional on a non-significant total effect have inflated type I errors. Next, we provide analytical evidence for those simulation findings by deriving large sample approximations for the relative power of the test for the indirect effect vs. the test for the total effect, in the absence of a direct effect. As those approximations both rely on the normality of the product of coefficients estimator of the indirect effect, which is known not to hold in small samples (Kisbu-Sakarya et al., 2014), simulation studies are performed. Inference for the indirect effect herein will not rely on asymptotic normality but on the bootstrap. Those simulations confirm the approximate analytical results for the circumstances under which higher power of the test for the mediated effect vs. the test for the total effect may indeed be expected.

**Figure 2:**
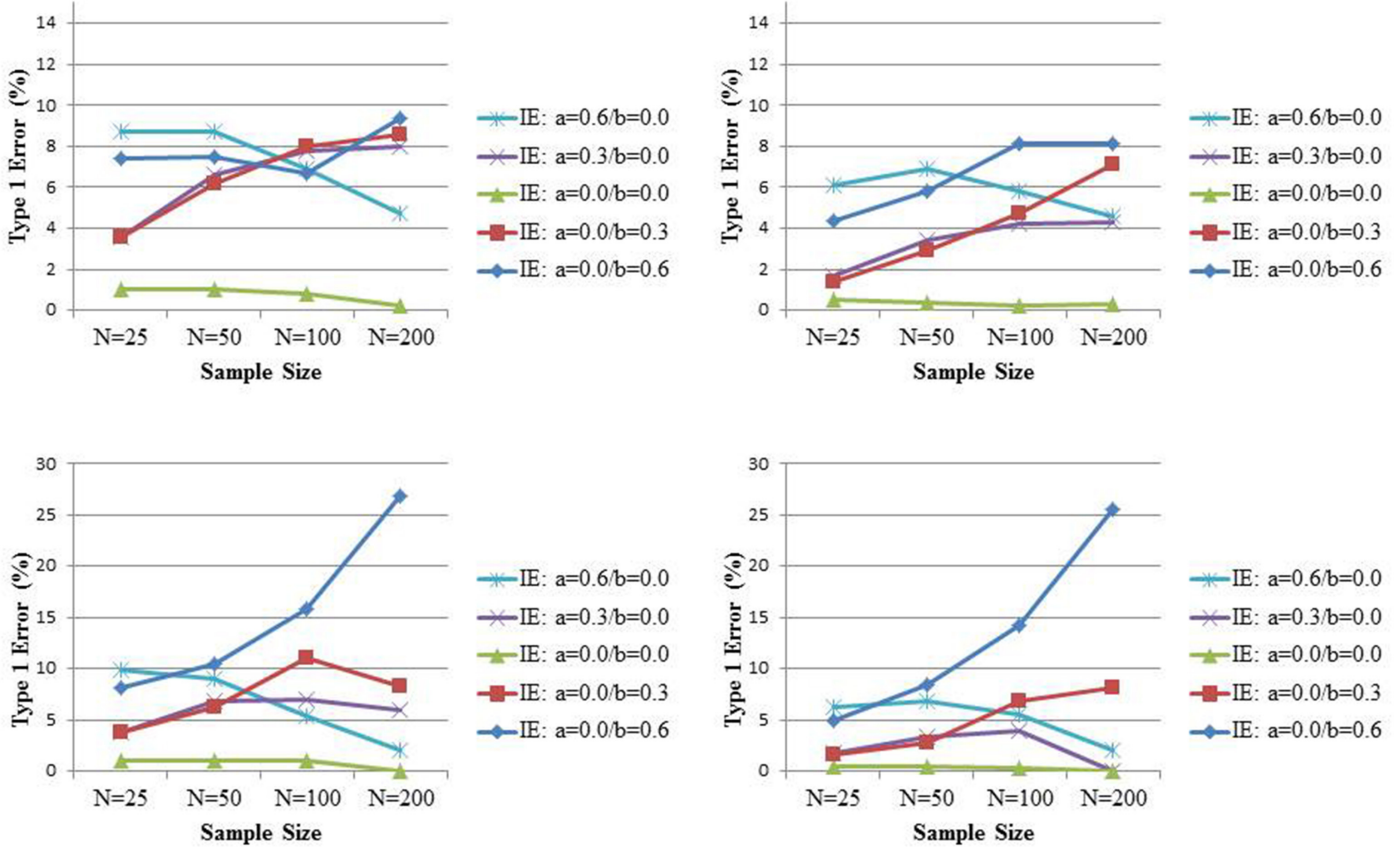
**The probability to reject the null hypothesis of no indirect effect (IE) when the total effect (TE) is not significant**. The true TE equals 0.20 (lower panel) and 0.30 (upper panel) while the IE equals 0 (with different combinations for its components, the path coefficients *a* and *b*). Significance is assessed at the 0.05 level and inference based on bias-corrected bootstrap intervals (left panel) or percentile bootstrap intervals (right panel).

While Rucker et al. (2011) explored the power to detect an indirect effect when no significant total effect is found, they did not investigate the Type I error of such strategy; that is, the probability to find a significant indirect effect in the absence of a significant total effect when there is no indirect effect. To this end, we simulate data under the assumption of a total effect equal to 0.2 or 0.3 but no indirect effect (because of the absence of an effect of *X* on *M*, or the absence of an effect of *M* on *Y*, or both) for varying sample sizes size *N* (25, 50, 100, 200). The variables *X*, *M*, and *Y* are all normally distributed with variance equal to 1, such that similar to Rucker et al. (2011), all population coefficients can be considered as standardized effects. For each combination of conditions, 5000 samples are generated. The total effect is estimated from Equation (1), while the indirect effect is estimated by the product of coefficients *a* and *b* from Equations (2) and (3). Significance of the indirect effect is based on its 95% bias-corrected bootstrap confidence (Hayes and Scharkow, 2013) or percentile-based bootstrap confidence intervals (Fritz et al., 2012). Bias-corrected bootstrap intervals are known to be somewhat too liberal in the simple linear setting we are considering, especially in smaller sample sizes (Hayes and Scharkow, 2013), and found to have the highest statistical power of the common tests of mediation. Fritz et al. (2012) cautioned researchers for equating the “most powerful” with “best” test, and found percentile bootstrap to be more accurate than bias-corrected bootstrap in terms of Type I error in the small samples we are considering. These authors argued that in practice one should decide a priori on the use of one of those tests based on whether avoiding Type I error or Type II error is of greater concern; for completeness we will present both here. The upper and lower panel of Figure 2 present the results for *c* equal to 0.2 and 0.3, respectively, with the bias-corrected and percentile bootstrap on the left and right side, respectively. In these graphs, the percentage of times that the indirect effect is declared to be significant while the total effect was not, is presented (that is, if in 2100 samples the total effect was not significant, the percentage is over these 2100 samples). Under either type of bootstrap, there is evidence that with increasing sample size and increasing total effect size, the Type I error for the indirect effect under such conditional approach gets seriously inflated, especially when the mediator is distal (i.e., *b* > *a*). Interestingly, the inflation is positively associated with the correlation between the test for the total effect and the test for the indirect effect (in our simulation setting this correlation equals *b*). These results suggest that power evaluations of tests for mediated effect after a non-significant effect has been found, should be considered with scrutiny because such tests come with inflated Type I errors.

We will now derive approximate analytic expressions for the relative power of the test for the indirect effect and the test for the total effect in the presence of complete mediation when using model (1) through (3). That is, we assume that the true value of *c*' equals zero in expression (3), but consider the linear regression Equations (2) and (3) for estimation of the indirect effect. Denoting the variance of *X*, ε_2_, and ε_3_ by σ^2^_X_, σ^2^_*M*|*X*_, and σ^2^_*Y*|*M*,*X*_, which are assumed to be constant here, it can easily be shown (MacKinnon, 2008, pp. 88–89) that in samples of size *n*, the variance of the estimated path coefficients *a* and *b* equal

$$Var\left(\hat{a}\right)=\frac{1}{n}\frac{\sigma_{M|X}^{2}}{\sigma_{X}^{2}}$$

$$Var\left(\hat{b}\right)=\frac{1}{n}\frac{\sigma_{Y|M,X}^{2}}{\sigma_{M|X}^{2}}$$

The Sobel-test (Sobel, 1982) that is frequently used to assess mediation, provides an approximate estimate for the standard error of $\hat{a}\hat{b}$. Using the (first-order) delta method for the variance of the estimated indirect effect, one finds the following expression

$$Var\left(\hat{a}\hat{b}\right)=b^{2}Var\left(\hat{a}\right)+a^{2}Var\left(\hat{b}\right)$$

The variance of the total effect, in the absence of a direct effect, equals

$$\begin{array}{l} Var\left(\hat{c}\right)=\frac{1}{n}\frac{\sigma_{Y|X}^{2}}{\sigma_{X}^{2}}\\ \;=\frac{1}{n}\frac{E\left(\sigma_{Y|M,X}^{2}|X\right)+Var\left(E\left(Y|X,M\right)|X\right)}{\sigma_{X}^{2}}\\ \;=\frac{1}{n}\frac{\sigma_{Y|M,X}^{2}+b^{2}\sigma_{M|X}^{2}}{\sigma_{X}^{2}} \end{array}$$

where the second equality follows from the law of iterated expectations, and the third equation from *Var*(*E*(*Y*|*X*,*M*)|*X*) = *Var*(*i*_3_ + *c*′*X* + *bM*|*x*) = *b*^2^σ^2^_*M*|*X*_. Wald-tests, based on $\hat{c}/\sqrt{Var\left(\hat{c}\right)}$ and $\hat{a}\hat{b}/\sqrt{Var\left(\hat{a}\hat{b}\right)},$, are commonly used to test for the total and indirect effect, respectively. Despite their common use, the normality of the indirect effect estimator is known to be flawed since the distribution of the product of two coefficients is not normal, and tests relying on the asymmetric distribution of the product or resampling procedures such as bootstrap are shown to be superior (MacKinnon et al., 2002). Recently, Kisbu-Sakarya et al. (2014) describe for which values of *a* and *b*, the deviation from normality for $\hat{a}\hat{b}$ is most severe, and how the moments of the indirect effect influence the coverage and imbalance of the Wald confidence intervals. Although we acknowledge this limitation of the Wald test for the indirect effect, we will use the ratio

(4) $$\left(\hat{a}\hat{b}/\sqrt{Var\left(\hat{a}\hat{b}\right)})/(\hat{c}/\sqrt{Var\left(\hat{c}\right)}\right)$$

as a proxy for the relative efficiency of the test of the indirect effect vs. the test of the total effect. We hereby assume that the power of the test for the indirect effect will be larger than the power of the test of the total effect, when the value of the Wald test statistic of the first is larger in absolute value than the value of the Wald test statistic of the latter. Expression (4) turns out to be helpful to make analytical progress with insightful results, but we caution the reader not to use the Wald test as a vehicle to perform power calculations. To the latter end, O'Rourke and MacKinnon (2014) provide analytical power expressions of the indirect effect relying on joint significance testing^1^, hereby avoiding the distribution of the product of coefficients. These authors also provide software programs to perform such calculations. Note however that these use partial correlations (MacKinnon, 2008, pp. 80–81) as effect size measures of individuals paths in the mediated effect whereas we consider standardized regression coefficients here.

In the case of complete mediation, the expected values of $\hat{a}\hat{b}$ and $\hat{c}$ are the same. When comparing the variance of the estimator of the indirect effect with the variance of the estimator of the total effect, we find that

$$\begin{array}{l} Var\left(\hat{a}\hat{b}\right)<Var\left(\hat{c}\right)\Leftrightarrow b^{2}\frac{\sigma_{M|X}^{2}}{\sigma_{X}^{2}}+a^{2}\frac{\sigma_{Y|M,X}^{2}}{\sigma_{M|X}^{2}}<\frac{\sigma_{Y|M,X+b^{2}\sigma_{M|X}^{2}}^{2}}{\sigma_{X}^{2}}\\ \;\Leftrightarrow \frac{a^{2}}{\sigma_{M|X}^{2}}<\frac{1}{\sigma_{X}^{2}}. \end{array}$$

Assuming standardized variables, we have that σ^2^_*X*_ = 1 and σ^2^_*M*|*X*_ = 1 − *a*^2^. Hence, using Equation (4) as a proxy of the relative efficiency, the power of the test for the indirect effect will be larger than the power of the total effect when *a*^2^/(1 – *a*^2^) < 1 or when *a* is smaller than $\sqrt{2}$/2 ≈ 0.70. This analytical finding is in line with the observation of Kenny and Judd (2014) based on their simulation study that “when *a* is very large (about 0.8 or higher), the excessive collinearity between X and M can result in the power of *c* to be greater than power of *ab*.” This point is also supported by O'Rourke and MacKinnon (2014) who showed that “when collinearity between X and M is high, the standard error of b is increased, leading to a less powerful test of significance.”

Interestingly, Cox (1960) showed that when immediately assuming no direct effect, i.e., considering regression model

$$Y=i_{4}+b_{c}\;M+\varepsilon_{4}$$

rather than Equation (3), the variance of the total effect is always larger than the variance of the indirect effect. That is, *Var*($\hat{a}\hat{b}$_*c*_) < *Var*($\hat{c}$), where *b_c_* reflects the effect of *M* on *Y* assuming complete mediation. Obviously, such modeling strategy would require strong prior knowledge about the absence of the direct effect of *X* on *Y*.

A second observation made by Kenny and Judd (2014) is that “the power advantage of testing *ab* over *c* declines as the mediator becomes either more proximal or more distal. Thus, the power advantage is greater when *a* and *b* are relatively equal.” This claim can also be formally derived by noting that the variance of the indirect effect is minimized when

$$a=\sqrt{\frac{{\left(ab\right)}^{2}-\sqrt{{\left(ab\right)}^{{}^{2}}-{\left(ab\right)}^{{}^{4}}}}{2{\left(ab\right)}^{{}^{2}}-1}}.$$

This expression follows from solving $\frac{\partial Var\left(\hat{a}\hat{b}\right)}{\partial a}=0$. Indeed, assuming standardized variables and complete mediation we have that σ^2^_*X*_ = 1, σ^2^_*M*|*X*_ = 1 − *a*^2^, and σ^2^_*Y*|*M*,*X*_ = 1 − *b*^2^, and thus

$$Var\left(\hat{a}\hat{b}\right)=\frac{1}{n}(b^{2}\left(1-a^{2}\right)+a^{2}\frac{1-b^{2}}{1-a^{2}}).$$

Taking the first derivative with respect to *a*, and setting this equal to zero, one finds (1 − 2*a*^2^*b*^2^)*a*^4^ + (2*a*^2^*b*^2^) *a*^2^ − *a*^2^*b*^2^ = 0. Solving this quadratic equation in *a*^2^ yields

$$a^{2}=\frac{{\left(ab\right)}^{2}-\sqrt{{\left(ab\right)}^{{}^{2}}-{\left(ab\right)}^{{}^{4}}}}{2{\left(ab\right)}^{{}^{2}}-1}.$$

This result was also shown without proof by Hoyle and Kenny (1999), but has a typo.

To see whether the above two analytical findings that naively relied on the normality of the indirect effect estimator also hold in small samples when no distributional assumptions about the indirect effect estimator are made, we elaborate the simulation study by Rucker et al. (2011). We focus here exclusively on settings with complete mediation (i.e., the direct effect *c'* is set to zero) and vary the coefficients *a* and *b* such that their product, and hence the indirect effect, equals 0.16 or 0.25 respectively, in samples of size *N* (25, 50, 100, 200). Based on the above calculations, the power for the test of the indirect effect is expected to be maximal when *a* ≈ 0.37 and *a* ≈ 0.45, under the settings where *ab* is equal to 0.16 and 0.25, respectively. Of note, Rucker et al. (2011) always assumed *a* = *b* in their simulation study and thus considered the nearly most beneficiary scenario for the power of the test for the indirect effect vs. the power for the test for the total effect. The variables *X*, *M*, and *Y* are again all normally distributed with variance equal to 1. For each combination of conditions, 5000 samples are generated. As before, the total effect is estimated from Equation (1), while the indirect effect is estimated by the product of coefficients *a* and *b* from Equations (2) and (3). To enable a fair comparison, 95% confidence intervals for both the total and indirect effect are both based on either bias-corrected or percentile bootstrap. As mentioned before, the first is known to be the most powerful approach for assessing indirect effects based on models (2) and (3).

**Figure 3:**
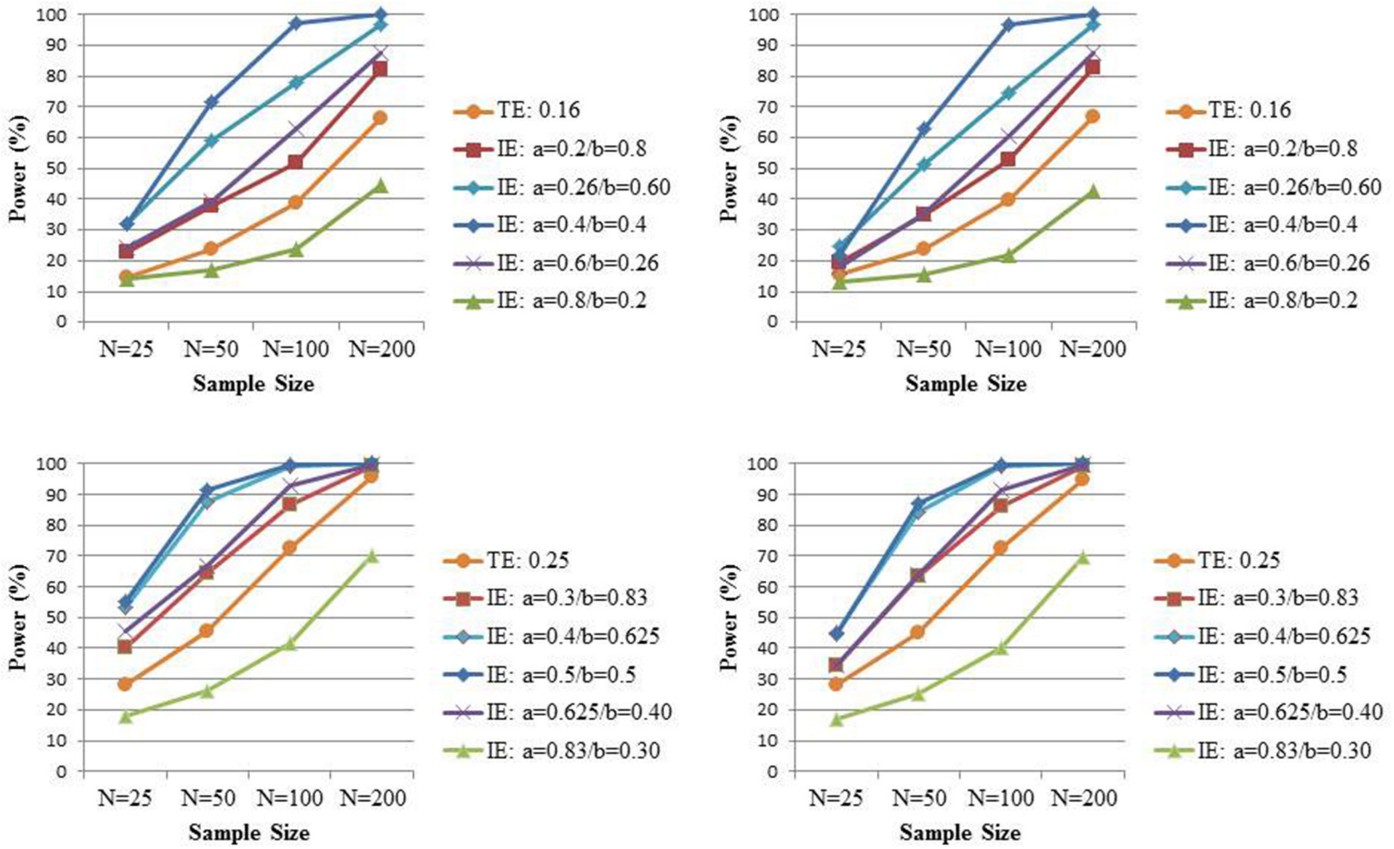
**The power to detect the total effect (TE) *c* and the power to detect the indirect effect (IE) *ab* under complete mediation when *c* = *ab* = 0.16 (upper panel) or *c* = *ab* = 0.25 (lower panel) for varying combination of *a* and *b* and varying sample sizes**. Significance is assessed at the 0.05 level and inference based on bias-corrected bootstrap intervals (left panel) or percentile bootstrap intervals (right panel).

The upper left panel of Figure 3 shows the power to detect at the 0.05 level an indirect effect equal to 0.16 for various combinations of the coefficients *a* and *b*, and the power to detect the total effect of the same size (the latter is averaged over the different combinations for the coefficients *a* and *b* as there were no relevant differences amongst the five different scenarios), while in the lower left panel both the indirect and total effect equal 0.25. The left panel presents the bias-corrected bootstrap and the right panel the percentile bootstrap. In contrast to Rucker et al. (2011)^2^, we prefer to present the marginal power for each test. That is, we look at the percentage of times a significant total effect is found (irrespective of the significance of the indirect effect) and the percentage of times a significant indirect effect is found (irrespective of the significance of the total effect), because this reflects the power of a procedure whereby one tests for indirect effect regardless of the test of total effect. In contrast, the power displayed in Rucker et al. (2011), for instance, reflects the power for a procedure whereby one only tests for mediated effect when a non-significant total effect is found; such procedure is not one that is normally adopted in practice. As predicted from the above analytical derivation, we indeed observe the highest power for the indirect effect when the standardized effect of *X* on *M* approximately equals the standardized effect of *M* on *Y*. But the power to detect the indirect effect is smaller than the power to detect the total effect of the same size when *a* is very large. Under the latter scenario, the high multicollinearity between *X* and *M* may result in imprecise estimators for *b* (Beasley, 2014; O'Rourke and MacKinnon, 2014). Note that those observations are confirmed with either type of bootstrap.

## Omitted M-Y confounders and the power of the test for the indirect effect

Claims about increased power for the test of an indirect or mediated effect are not only somewhat misguided by the inflated type I error, but also because the increased power is merely the result of making more assumptions: model assumptions on the joint distribution of outcome and mediator, and structural assumptions on the absence of common causes. Indeed, to identify the indirect effect one needs to assume the absence of no unmeasured confounders of the mediator-outcome relationship. Technically, this implies no correlation between the error terms ε_2_ and ε_3_ in models (2) and (3). In practice however, there will typically be variables that affect both the mediator and outcome but that are not measured or controlled for. Therefore, an apparent indirect effect may be (partially) driven by a spurious correlation between *M* and *Y* shows the extreme scenario where the effect of *M* on *Y* is completely due to the omitted variable *U*.

**Figure 4:**
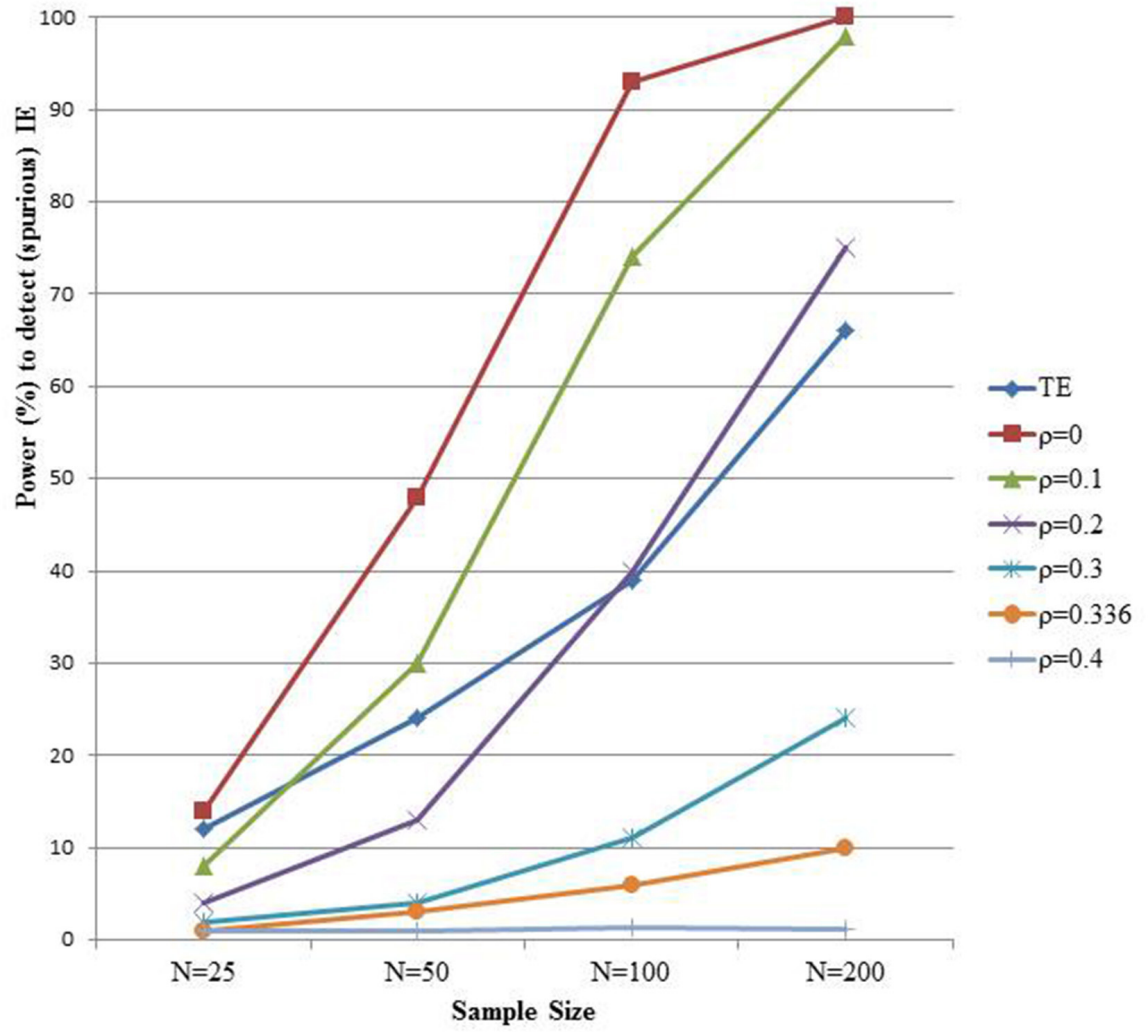
**The power to detect the indirect effect (IE)**. Data are generated according to the right panel of Figure 1 with *a* = 0.4 and *c*' = 0.16 and a residual correlation rho between M and Y equal to 0.336. The different power curves represent varying assumptions on unmeasured confounding of the M-Y relationship in a sensitivity analysis.

We mimic the setting of Rucker et al. (2011) with the direct effect equal to 0.16, the effect of *X* on *M* fixed to 0.4, but now with a spurious correlation between *M* and *Y* induced by a standard normal distributed variable *U* (the right panel of Figure 1). Typically, factors other than *X* that affect *M* also affect *Y* in the direction that *M* affects *Y* (Bullock et al., 2010). We assume here that *U* has the same effect on *M* and *Y*, and results in a spurious 0.4 effect of *M* on *Y*. Hence, the true total and indirect effect equal 0.16 and 0, respectively; but the spurious indirect effect, ignoring the unmeasured *U*, also equals 0.16. We simulate such data in samples of size *N* (25, 50, 100, 200), and repeated each setting 5000 times. The total and indirect effect are estimated each time using models (1), (2), and (3). Figure 4 shows the power to detect the total effect, as well as the power to detect the spurious indirect effect (curve with ρ equal to zero, cfr. infra). As expected, one finds as before substantial power gain for the test of *ab* vs. *c* with increasing sample size. However, this is misguided in view of the absence of an indirect effect.

One may wonder what the value of the estimated indirect effect would be if one allows for unmeasured *M*-*Y* confounding. Sensitivity analyses can shed some light on the robustness of indirect effect estimates against such violations. The *mediation* R-package of Tingley et al. (2014) provides a convenient sensitivity analysis by assessing the indirect effect for varying levels of correlation ρ between ε_2_ and ε_3_ (Imai et al., 2010). If there were no unmeasured confounders such as *U* in the right panel of Figure 1, then ρ equals zero. In the above described simulation setting the true correlation ρ equals 0.366. The different curves in Figure 4 show the probability to detect a significant indirect effect for varying levels of the sensitivity parameter ρ. With increasing values of ρ, and hence smaller estimated indirect effect, the gain in power of the test for the indirect effect vs. the power of the total effect disappears. This is even more pronounced if one considers that, since ρ is unknown, the presence of an indirect effect can in principle only be concluded when there is significant evidence for it at all possible values of ρ.

In practice, one must thus face the uncertainty about the no unmeasured confounding assumption for the *M*-*Y* relationship when assessing the indirect effect. Because that assumption is not needed for the total effect, one may argue that some penalty should be paid for the test of the indirect effect vs. the test of the total effect by allowing an amount of unmeasured *M*-*Y* confounding. The extent of that penalty must reflect the uncertainty about the no unmeasured confounding assumption for the *M*-*Y* relationship. Expert knowledge for the particular study at hand is needed for this purpose. Because we observed higher power for the test of the indirect vs. the total effect under most scenarios, it is informative to explore which value of the sensitivity parameter ρ makes the power gain of the test for *ab* vs. the test for *c* disappear. This is shown for a particular setting in Figure 4, but we perform such calculation for each of the settings in the simulation study presented in Figure 2. We therefore varied the values of the sensitivity parameter ρ in the analyses, and estimated each time the corresponding indirect effect and its standard error. The value of ρ that makes the power of the test for the indirect and the test for the total effect equal is presented in Table 1 for each of the settings. Interestingly, we observe that when *a* = *b* rather than *b* > *a*, smaller values of ρ suffice to lose the gain in power of the test for the indirect effect vs. the test for the total effect. Hence, in those situations where the largest gain in power is observed, weaker violation of the unmeasured confounding assumption suffices to end up with equal power to detect the indirect and total effect.

**Table 1:** **Value of the sensitivity parameter ρ that makes the power of the test for the indirect effect and the power of the test for the total effect equal**.

	***N* = 25**	***N* = 50**	***N* = 100**	***N* = 200**
**INDIRECT EFFECT = 0.16**				
*a* = 0.20/*b* = 0.80	0.15	>0.50	>0.50	>0.50
*a* = 0.26/*b* = 0.60	0.07	0.30	0.41	0.46
*a* = 0.40/*b* = 0.40	0.03	0.16	0.20	0.22
*a* = 0.60/*b* = 0.26	0.01	0.06	0.04	0.06
**INDIRECT EFFECT = 0.25**				
*a* = 0.30/*b* = 0.83	>0.50	>0.50	>0.50	>0.50
*a* = 0.40/*b* = 0.63	0.16	0.36	0.40	0.40
*a* = 0.50/*b* = 0.50	0.11	0.20	0.22	0.22
*a* = 0.63/*b* = 0.40	0.05	0.12	0.13	0.14

Note that we focused above on the assumption of no unmeasured *M*-*Y* confounders, but other assumptions such as “there are no measured confounders of the *M*-*Y* relationship affected by *X*” are needed too to identify the mediated effect. We refer the interested reader to a Imai et al. (2010) and Pearl (2014) for more details on the identification assumptions for direct and indirect effects.

## Practical implications

What are the practical implications of the above findings? We argue that much depends on the research hypotheses specified at the design of the study. Does the study primarily aim to establish the total effect of an intervention on an outcome (“the total effect approach”) or is the focus solely on the underlying processes (“the mediation only approach”). Under the total effect approach, the first question that should be raised concerns the power of the study to detect a relevant total effect. Only a small proportion of articles in social sciences contain a solid description of power and/or sample size calculations that were made *before* any data were collected. If a sufficiently powered study fails to detect a total effect, should we start looking at one or more mediators? First, we concur with O'Rourke and MacKinnon (2014) that the inclusion of a mediator that occurs *post-hoc* because of failure to find a significant total effect is inappropriate but that such inclusion should be specified at the design stage. Second, we have shown in this paper that the type I error of a strategy whereby one stops testing after a significant total effect but continues to test for mediation after a non-significant total effect may be highly inflated. One potential solution would be to apply some multiple testing correction. However, since the test for the indirect effect and total effect are dependent (Tofighi et al., 2009), such correction is not straightforward. A conservative approach would be to apply some type of Bonferroni correction. Third, even if one would correct somehow for this inflation, one should realize that the potential higher power of the test of the indirect effect vs. the test of the total effect rests on strong untestable assumptions. Alternatively, one may argue that unknown suppression mechanisms may come into play and explain the lack of total effect. MacKinnon et al. (2000) use the term suppressor to describe “a variable which increases the predictive ability of another variable by its inclusion in a regression equation.” Suppression occurs when the indirect effect via the suppressor has an opposite sign to that of the total effect, and thus its omission might lead to the total effect to appear small or non-significant. Not seldom, papers presenting significant indirect effects in the absence of a significant total effect show (possibly non-significant) direct and indirect effects with opposite signs resulting in a small total effect. Simultaneously examining multiple mediators (with or without opposite indirect effects) may reveal further insights, but such explorations are typically *post-hoc* or cannot even be performed because the potential suppressor is not measured. Moreover, one should be aware of the strong assumptions needed to identify the indirect effects for each mediator separately in such multiple mediator models (Imai and Yamamoto, 2013).

Under the mediation only approach on the other hand, there is no need to explicitly test for the total effect but it should be made clear in the reporting of such studies that the identification of the indirect effect requires stronger assumptions than the total effect. At the design stage, researchers should not only perform an adequate sample size calculation to detect indirect effects with sufficient power (Fritz and MacKinnon, 2007), but also think carefully about common causes of mediator and outcome. At the analysis stage, one should then consider models that control for those measured potential confounders of the *M*-*Y* relationship. Additionally, we plea for sensitivity analyses (Imai et al., 2010; Tingley et al., 2014) to be standard part of mediation analyses. How robust is the finding of a significant indirect effect against violations of the no unmeasured *M*-*Y* confounder assumption? If a small value of the sensitivity parameter(s) makes the indirect effect insignificant, results may not be fully convincing. In this paper, we used sensitivity analyses along the lines of Imai et al. (2010) and considered the correlation ρ between ε_2_ and ε_3_ as the sensitivity parameter. The latter only requires a single sensitivity parameter, but may be intuitively hard to understand. Alternative approaches have recently been proposed to assess indirect effect bias. Cox et al. (2014) for example build on the LOVE (left out variables error) method by Mauro (1990) and assess omitted variable bias for the mediator-outcome relation using two correlations: (i) the correlation between a hypothesized confounder and the outcome and (ii) the correlation between this confounder and the mediator. These correlations are intuitively easier to understand and their approach also enables the researcher to identify the different combinations of sizes of these correlations that cause the indirect effect to become zero.

In summary, whereas other scholars (Rucker et al., 2011; Kenny and Judd, 2014; O'Rourke and MacKinnon, 2014) mostly discussed the power of the test for the indirect effect vs. the power of the test for the total effect, we shifted the focus to the type I error and the impact of unmeasured *M*-*Y* confounding. When mediation is of primary interest, we suggest on the one hand to build in some conservatism by pleading for sensitivity analyses as a compulsory part of every mediation analysis, but on the other hand to drop the prerequisite of a significant total effect. First, since there is no single method that can deal with unmeasured *M*-*Y* confounding given the observed data, unless other strong untestable assumptions are made (MacKinnon and Pirlott, 2014), we view sensitivity analyses as a necessity. Second, by dropping the requirement of a significant total effect on the other hand, we may decrease the type II error (i.e., missing true mediated effects) that is associated with the causal steps approach (MacKinnon et al., 2002). Most of the arguments that we discussed above are exacerbated in non-randomized studies. But precisely because randomization simplifies the assumptions, the randomized experiment was the proper framework for highlighting complications. We hope authors and reviewers find the above guidelines useful to assess the trustworthiness of an estimated indirect effect in future publications.

### Conflict of interest statement

The authors declare that the research was conducted in the absence of any commercial or financial relationships that could be construed as a potential conflict of interest.

## Footnotes

^1^One way to test the null hypothesis that *ab* = 0 is to test that both paths *a* and *b* are zero. Simulation results have shown that this test performs about as well as bootstrap (Hayes and Scharkow, 2013). Its major drawback is that it does not provide a confidence interval for the indirect effect.

^2^These authors claimed to present the power to detect a significant indirect effect in the absence of a significant total effect (i.e., a conditional power), but actually showed the joint probability of a significant indirect effect and a non-significant total effect.
